# Cyclic cryotherapy with vitamin D facilitates early rehabilitation after total knee arthroplasty

**DOI:** 10.3389/fmed.2024.1380128

**Published:** 2024-04-29

**Authors:** Fulin Li, Yingrong Mo, Xiao Huang, Ke Sun, Baichuan Li, Dong Yin

**Affiliations:** ^1^Department of Joint Surgery and Sports Medicine, The People's Hospital of Guangxi Zhuang Autonomous Region, Guangxi Academy of Medical Sciences, Nanning, China; ^2^Department of Pharmacy, The People's Hospital of Guangxi Zhuang Autonomous Region, Guangxi Academy of Medical Sciences, Nanning, China

**Keywords:** vitamin D, cyclic cryotherapy, total knee arthroplasty, rehabilitation, satisfaction

## Abstract

**Objective:**

This study aimed to evaluate the efficacy of cyclic cryotherapy and vitamin D administration on early rehabilitation after total knee arthroplasty (TKA), as its efficacy remains unclear.

**Methods:**

We divided 150 patients (three groups) who underwent TKA into those treated with or without cyclic cryotherapy and vitamin D.

**Results:**

Compared with patients who did not receive cyclic cryotherapy, those who received postoperative cyclic cryotherapy and vitamin D supplementation had significantly higher American Knee Society Scores (AKSS) on postoperative day (POD) 7 and at 1 month postoperatively; higher visual analogue scale (VAS) values on POD1–3 and POD7; reduced thigh swelling on POD3 and POD7; increased range of motion (ROM) on POD3, POD7, and at 1 month postoperatively; and reduced postoperative length of stay (PLOS). However, no significant difference in patient satisfaction was observed between the patient groups. At 1 and 3 months postoperatively, patients administered cyclic cryotherapy and vitamin D had significantly higher AKSS, ROM, and vitamin D levels than those who did not receive vitamin D. No perioperative complications such as surgical site infection, skin frostbite, or vitamin D intoxication were observed.

**Conclusion:**

Cyclic cryotherapy post-TKA had short-term advantages in terms of AKSS, VAS, thigh swelling, ROM, PLOS, and accelerated rehabilitation, but did not improve patient satisfaction. Cyclic cryotherapy combined with vitamin D improved AKSS and ROM at 1 and 3 months postoperatively.

## 1 Introduction

Knee osteoarthritis (KOA) is a common disease, which is often with local pain, joint deformity, swelling and motor dysfunction. In the late stage of KOA, it would greatly reduce the quality of life. Total knee arthroplasty (TKA) is an effective method for treating end-stage knee joint disease. With considerable progress in medical technology, TKA is widely used (1, 2). Owing to extensive soft tissue dissection, osteotomy, and other surgical interventions during TKA, blood loss, pain, swelling, and poor functional rehabilitation remain important factors hindering efficient rehabilitation in TKA (3, 4).

Some studies have reported that cryotherapy applied to soft tissue injuries successfully reduces early local limb pain, swelling, and the inflammatory response, and promotes early rehabilitation during TKA (5, 6). However, few studies have investigated the efficacy of cyclic cryotherapy post-TKA and its clinical efficacy remains unclear (7–9). Vitamin D (Vit D) has previously been shown to affect bone, muscle, and immunological function through a variety of mechanisms. Vit D deficiency is a common nutritional deficiency, which increases the risk of hip and non-vertebral fractures, muscle weakness, and the risk of falls (10, 11). Recently, the role of Vit D has been examined in relation to improving osteoarthritis (OA), perioperative pain, and muscle rehabilitation (12, 13). However, its role in improving functional rehabilitation post-TKA, which has been rarely reported, has not been established (14, 15). The effects of cold therapy are early, whereas the effects of vitamin D are slow and long-lasting, and they have different mechanisms. Whether there is a synergistic effect between a combination of cryotherapy and Vit D administration post-TKA to further accelerate postoperative rehabilitation and improve function is yet to be elucidated. Therefore, we aimed to evaluate the efficacy of applying cyclic cryotherapy and administering Vit D on early rehabilitation post-TKA.

## 2 Methods

### 2.1 Patients and design

This study was undertaken at the People’s Hospital of Guangxi Zhuang Autonomous Region (Guangxi Academy of Medical Sciences) from December 2017 to March 2022, and was approved by the Hospital Ethics Committee. The study was designed as a single-blind, prospective randomized controlled trial (Chinese Clinical Trial Registry No.: ChiCTR2300071161; registration date, 06/05/2023).

The inclusion criteria comprised patients who had undergone unilateral TKA for KOA with a pre-operative Vit D level < 75 nmol/L and who had provided their written informed consent prior to participation in the study. Exclusion criteria comprised patients with an allergy to Vit D and taking Vit D before surgery, or those unable to tolerate cryotherapy; and those with a history of severe heart disease (NYHA >2), liver or kidney failure, haematological diseases, diabetes mellitus, tumour, or systemic rheumatic diseases (rheumatoid arthritis, ankylosing spondylitis, or systemic lupus erythematosus). We also excluded patients with a prior history of knee surgery, preoperative venous thrombosis or vasculitis, severe osteoporosis; those with a lack of cognitive function and normal sensation, and those lost to follow-up.

The enrolled study patients were randomized into one of three groups (A, B, or C). The sequence to which they were randomly assigned was concealed in opaque sealed envelopes and opened only on the day before surgery.

During TKA, all patients were administered infused tranexamic acid (TXA, 1 g) 30 min prior to tourniquet deflation. TXA (1 g) was dissolved in normal saline (10 mL) and injected into the joint cavity prior to closing the incision. Patients in group A did not receive cryotherapy or Vit D. Patients in group B were treated with intermittent compression cyclic cryotherapy postoperatively for 30 min. Intermittent compression cyclic cryotherapy was administered four times daily (30 min each time) and continued until postoperative day (POD)7. In addition to the same treatment provided to group B, patients in group C were administered Vit D (intramuscular injection, 1 mL: 5 mg, 200,000 U, Haixin, Gannan, Jiangxi) on POD1, and a daily oral administration of 800 U (Xingsha, Xiamen, China) was continued for a further 3 months.

### 2.2 Surgery management

All TKAs were performed by senior physicians (the same team) in a 100-level laminar flow operating room. All patients were evaluated by an anaesthesiologist and were administered intravertebral anaesthesia in the supine position. All patients underwent a median incision approach and received a posterior stabilised prosthesis fixed with bone cement, and the patella was not replaced, and the infrapatellar fat was preserved in all patients. All patients were treated with the same prosthesis supplied by the same company and manufactured by Johnson & Johnson, United States. Following prosthesis implantation, the tourniquet was routinely released for wound hemostasis.

### 2.3 Postoperative care protocol

Ankle dorsi- and plantarflexion and quadricep strength exercises were started in the recovery bay. All patients received oral rivaroxaban (10 mg) once drainage was removed at 48 h postoperatively. Patients received standard supervised physiotherapy daily, including continuous passive motion, strength training, and walking. All patients received the same analgesia (Parecoxib and celecoxib, Pfizer Inc.). After returning to the ward, pain was assessed using a visual analogue scale (VAS) with scores ranging from 0 (no pain) to 10 (worst pain imaginable). Evaluations for detecting deep vein thrombosis were undertaken using Doppler ultrasonography pre-discharge and at 1 month postoperatively.

### 2.4 Outcome assessment

We used the American Knee Society Score (AKSS) (16) on POD7, and at 1 and 3 months postoperatively. The VAS was used on POD1–POD3 and on POD7. We evaluated the difference in thigh circumference on POD3 and POD7. We measured knee joint range of motion (ROM) on POD3, POD7, and at 1 and 3 months postoperatively. Postoperative length of stay (PLOS), the degree of patient satisfaction, and the level of Vit D (Roche cobas e 411/e 601 automatic electrochemiluminescence immunoassay) were determined on POD1, POD7, and at 1 and 3 months postoperatively.

The AKSS includes knee joint activity and function scores, with a maximum score of 200. The higher the score, the better the knee function and joint activities.

### 2.5 Patient satisfaction

A Quality of Recovery-40 questionnaire (QoR-40), which has been externally validated, was used to assess patient satisfaction following major surgery. It has five recovery criteria, namely, physical comfort, emotional state, physical independence, psychological support, and pain, and is graded from 40 to 200 (40 indicates poor patient satisfaction, 41–119 indicates minimal patient satisfaction, 120–159 indicates good satisfaction, and ≥ 160 indicates excellent patient satisfaction) (17).

### 2.6 Postoperative complications

Patients were evaluated for complications such as surgical site infections, skin frostbite, deep vein thrombosis, or Vit D intoxication. Surgical site infections were mainly assessed in relation to the condition of the incision and inflammatory indicators. Frostbite mainly manifested as cold, pale, and hard skin tissue, and numbness or loss of sensation in the affected area. The clinical manifestations of deep vein thrombosis mainly included pain and swelling in the calf region, and all patients were re-examined using B-mode ultrasound imaging of the lower limbs postoperatively. The main clinical manifestations of Vit D poisoning are loss of appetite, anorexia, irritability, emotional distress, lethargy, and low-grade fever. We monitored each patient’s Vit D levels during the follow-up period. We planned to stop Vit D supplementation immediately if a patient was observed to have a Vit D level above the normal level.

### 2.7 Statistical analyses

All statistical analyses were performed using SPSS.24 (IBM Corp., Armonk, NY, United States) software. Results are presented as mean ± standard deviation (for continuous variables) and number (for qualitative variables). One-way ANOVA and Tukey’s post-hoc tests were used to evaluate parametric data, and a Mann–Whitney U-test was used for nonparametric data. Pearson’s chi-square or Fisher’s exact tests were used to analyse qualitative comparative data. A *p*-value <0.05 was considered statistically significant. Based on a power of 0.90 and a significance level of 0.05, 43 patients per treatment group were required for this study. Considering the dropout rate, the sample size was increased from 10 to 15%. Therefore, a sample size of 50 patients in each group was required, with a total sample size of 150 patients (17).

## 3 Results

### 3.1 Patient demographics

**Figure 1:**
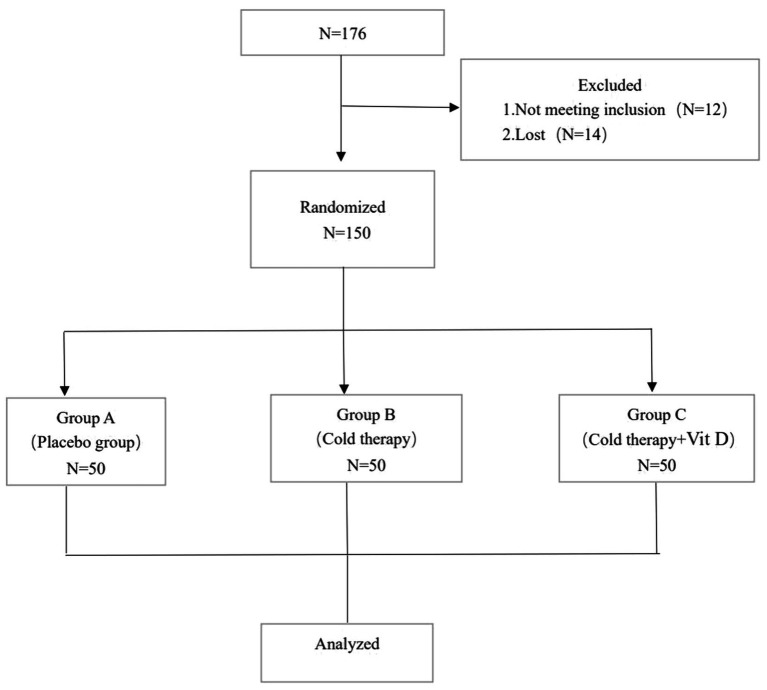
Schematic diagram of the patient study process.

A total of 176 patients were recruited between December 2017 and March 2022. All of the recruited patients were scheduled to undergo primary unilateral TKA at our medical institution. Of these, 12 patients did not meet the inclusion criteria, and 14 patients could not complete follow-up. The remaining 150 eligible patients were included in the intervention as part of three randomized groups (groups A-C, 50 participants per group) (Figure 1). Baseline characteristics and preoperative variables were comparable among the three groups (Table 1).

**Table 1:** Demographic data of the patients receiving TKA.

	Group A	Group B	Group C	F/χ^2^	*p*
*N*	50	50	50	–	–
Gender (M/F)	16/34	15/35	18/32	0.42	0.81
Age (y)	67.18 ± 3.69	68.60 ± 6.70	68.98 ± 5.50	1.52	0.22
BMI (kg/m^2^)	25.53 ± 2.90	25.61 ± 2.72	25.89 ± 3.83	0.18	0.84
Operating time (min)	125.26 ± 13.47	121.62 ± 12.88	123.44 ± 13.77	0.93	0.40
Preoperative AKSS	86.69 ± 7.73	87.68 ± 7.60	86.44 ± 6.46	0.40	0.67
Preoperative VAS	7.62 ± 0.64	7.46 ± 0.65	7.46 ± 0.68	1.00	0.37
Preoperative ROM (°)	86.40 ± 7.10	87.64 ± 7.43	87.04 ± 5.14	0.44	0.65
Preoperative Vitamin D	53.10 ± 7.32	53.66 ± 8.03	55.22 ± 9.14	0.90	0.41

BMI, body mass index; AKS, American knee society; VAS, visual analogue scale; ROM, range of motion.

### 3.2 Functional rehabilitation

**Figure 2:**
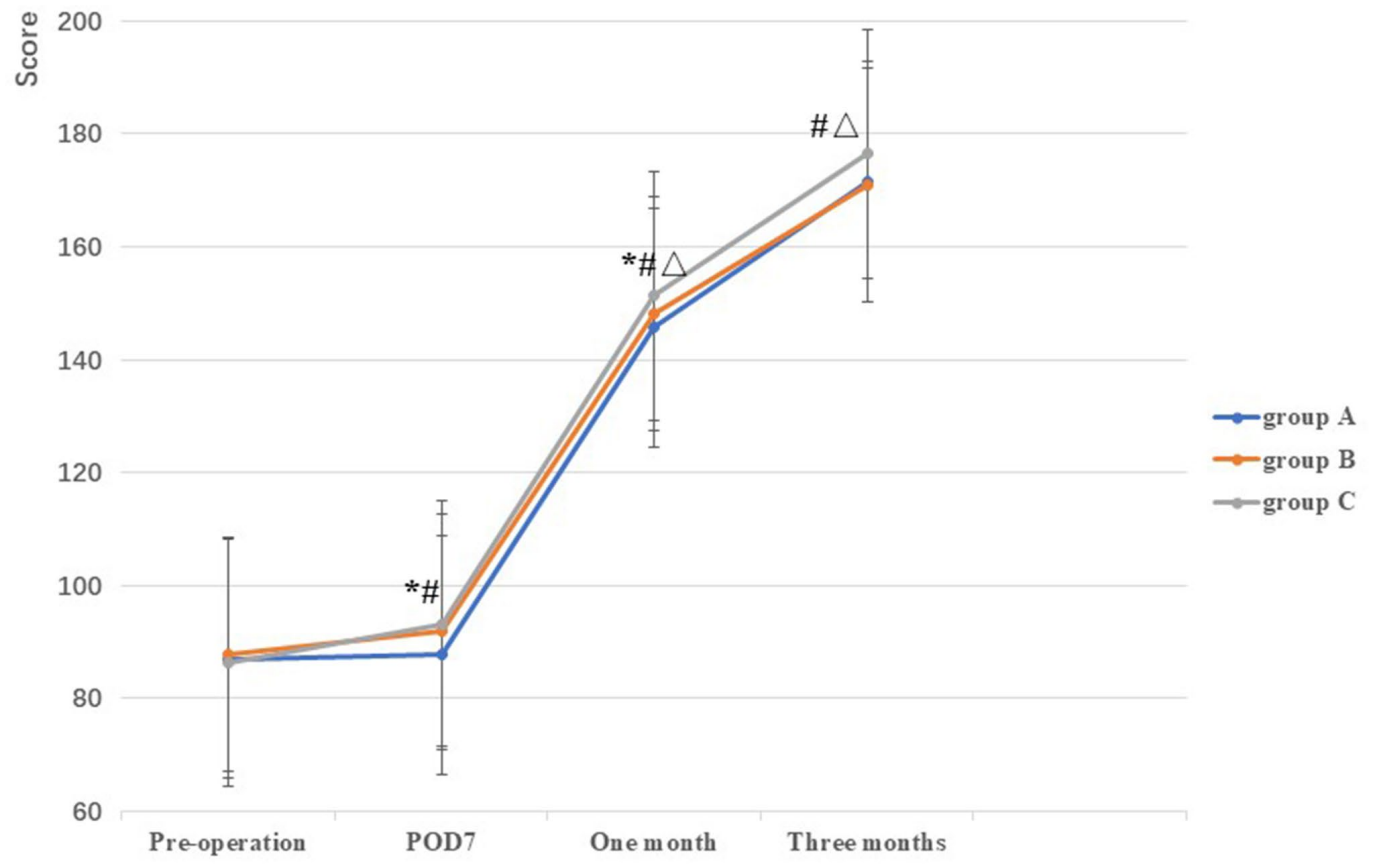
Comparison of AKSS among the three groups at different stages. *: A vs B, *p*<0.05; #: A vs C, *p*<0.05, Δ: B vs C, *p*<0.05.

No significant difference in AKSS among the three groups was observed prior to TKA (*F* = 0.38, *p* = 0.69). Compared with the AKSS in group A on POD7 and at 1 month postoperatively (87.70 ± 5.17 and 145.64 ± 5.76, respectively), the AKSS in groups B (92.06 ± 3.84 and 148.24 ± 5.50, respectively) and C (93.08 ± 4.43 and 151.30 ± 4.45, respectively) were higher, and the difference was statistically significant (all *p* < 0.001). The AKSS in group C at 1 and 3 months postoperatively (151.30 ± 4.45 and 176.50 ± 3.78, respectively) was higher than that in groups A (145.64 ± 5.76 and 171.48 ± 6.23, respectively) and B (148.24 ± 5.50 and 170.98 ± 4.90, respectively), and the difference was statistically significant (all *p* < 0.05). However, no statistically significant difference was observed between groups A and B at 3 months postoperatively (*p* = 0.62) (Table 2; Figure 2).

**Table 2:** The clinical effect among the three groups.

	Group A	Group B	Group C	F	*p*	*P_AB_*	*P_AC_*	*P_BC_*
AKSS								
POD7	87.70 ± 5.17	92.06 ± 3.84	93.08 ± 4.43	20.05	0.00	0.00	0.00	0.26
One month	145.64 ± 5.76	148.24 ± 5.50	151.30 ± 4.45	14.46	0.00	0.015	0.00	0.004
Three months	171.48 ± 6.23	170.98 ± 4.90	176.50 ± 3.78	18.82	0.00	0.62	0.00	0.00
VAS								
POD1	7.76 ± 0.59	6.60 ± 0.67	6.68 ± 0.79	44.07	0.00	0.00	0.00	0.56
POD2	4.32 ± 0.78	2.96 ± 0.67	3.08 ± 0.63	59.12	0.00	0.00	0.00	0.39
POD3	2.68 ± 0.59	2.08 ± 0.57	2.06 ± 0.79	14.40	0.00	0.00	0.00	0.88
POD7	2.20 ± 0.57	1.66 ± 0.63	1.54 ± 0.65	16.33	0.00	0.00	0.00	0.33
Difference in circumference (cm)								
POD3	4.50 ± 0.49	4.21 ± 0.48	4.23 ± 0.32	6.96	0.00	0.001	0.002	0.82
POD7	3.98 ± 0.32	3.72 ± 0.33	3.70 ± 0.31	12.09	0.00	0.00	0.00	0.83
ROM (°)								
POD3	50.88 ± 3.43	54.32 ± 4.96	54.86 ± 5.41	10.65	0.00	0.00	0.00	0.57
POD7	71.70 ± 3.07	74.56 ± 5.15	74.60 ± 5.50	6.27	0.00	0.003	0.002	0.97
One month	93.52 ± 2.61	95.42 ± 3.49	98.26 ± 3.10	29.83	0.00	0.003	0.00	0.00
Three months	113.64 ± 3.12	113.92 ± 2.61	118.86 ± 2.39	58.07	0.00	0.61	0.00	0.00
PLOS	10.76 ± 0.89	10.12 ± 0.56	10.12 ± 0.59	16.60	0.00	0.00	0.00	0.47
Vitamin D (nmol/L)								
POD1	43.90 ± 8.06	44.10 ± 7.47	45.90 ± 8.06	0.98	0.38	0.90	0.21	0.25
POD7	40.22 ± 6.85	40.18 ± 6.82	42.88 ± 7.11	2.49	0.09	0.98	0.06	0.05
One month	44.56 ± 7.53	44.30 ± 7.20	61.34 ± 6.86	91.91	0.00	0.86	0.00	0.00
Three months	45.52 ± 6.69	45.14 ± 6.93	84.20 ± 5.44	617.14	0.00	0.77	0.00	0.00

AKSS, American knee society score; POD, postoperative day; ROM, range of motion; PLOS: postoperative length of stay. All *p* = 0.00 means *p* < 0.001.

**Figure 3:**
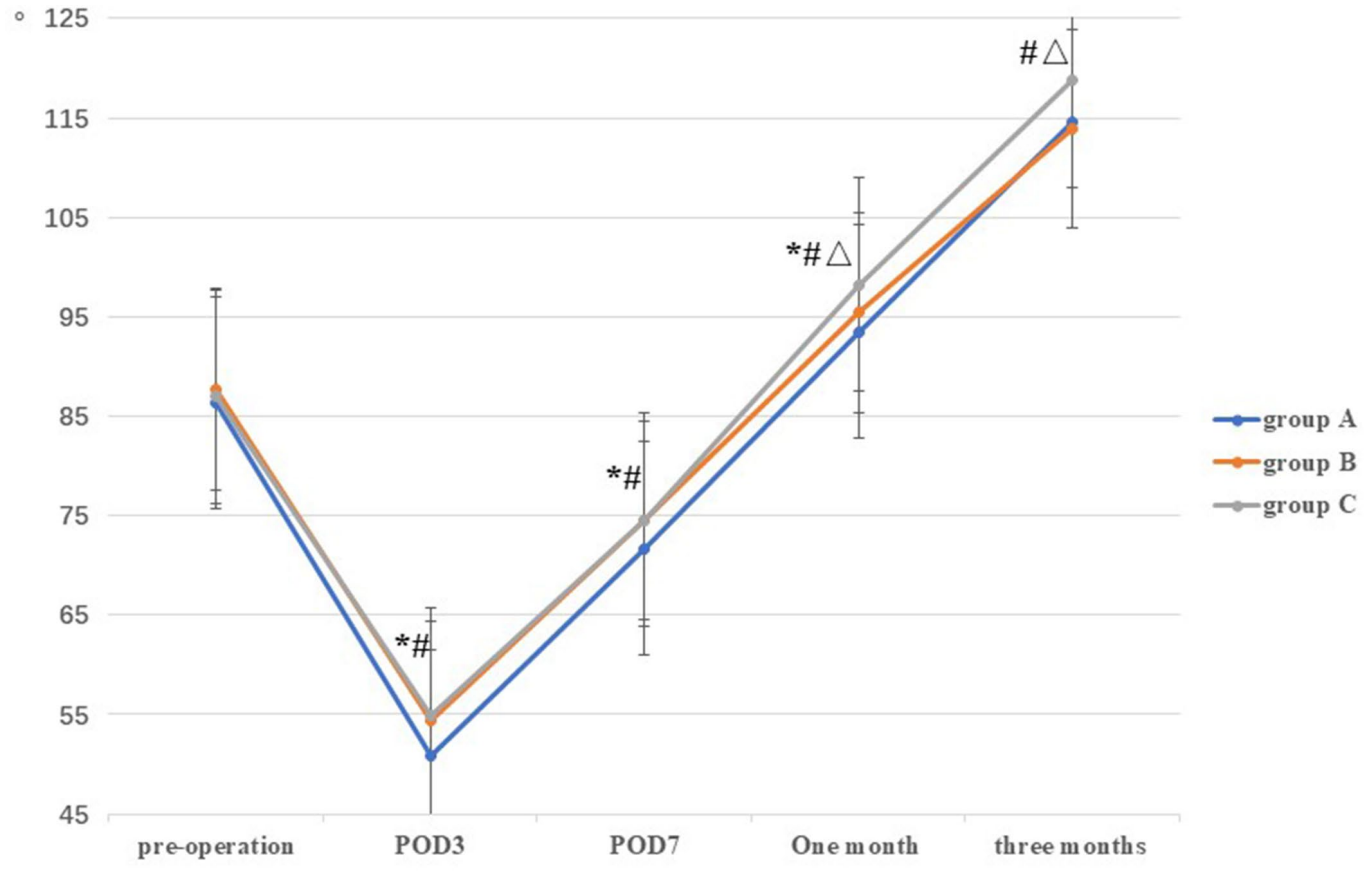
Comparison of ROM among the three groups at different stages. *: A vs B, *p*<0.05; #: A vs C, *p*<0.05, Δ: B vs C, *p*<0.05.

There was no significant differences in ROM among the three groups prior to TKA (*p* = 0.65). Compared with group A on POD3, POD7, and at 1 month postoperatively (50.88 ± 3.43, 71.70 ± 3.07, and 93.52 ± 2.61, respectively), the ROM in groups B (54.32 ± 4.96, 74.56 ± 5.15, and 95.42 ± 3.49, respectively) and C (54.86 ± 5.41, 74.60 ± 5.50, and 98.26 ± 3.10, respectively) were higher, and the difference was statistically significant (all *p* < 0.05). However, no statistically significant difference was observed between groups B and C on POD3 and POD7 (*p* > 0.05). At 1 and 3 months postoperatively, the ROM in group C (98.26 ± 3.10 and 118.86 ± 2.39, respectively) was higher than that in the groups A (93.52 ± 2.61 and 113.64 ± 3.12, respectively) and B (95.42 ± 3.49 and 113.92 ± 2.61, respectively), and the difference was statistically significant (all *p* < 0.05) (Table 2; Figure 3).

### 3.3 Pain level

**Figure 4:**
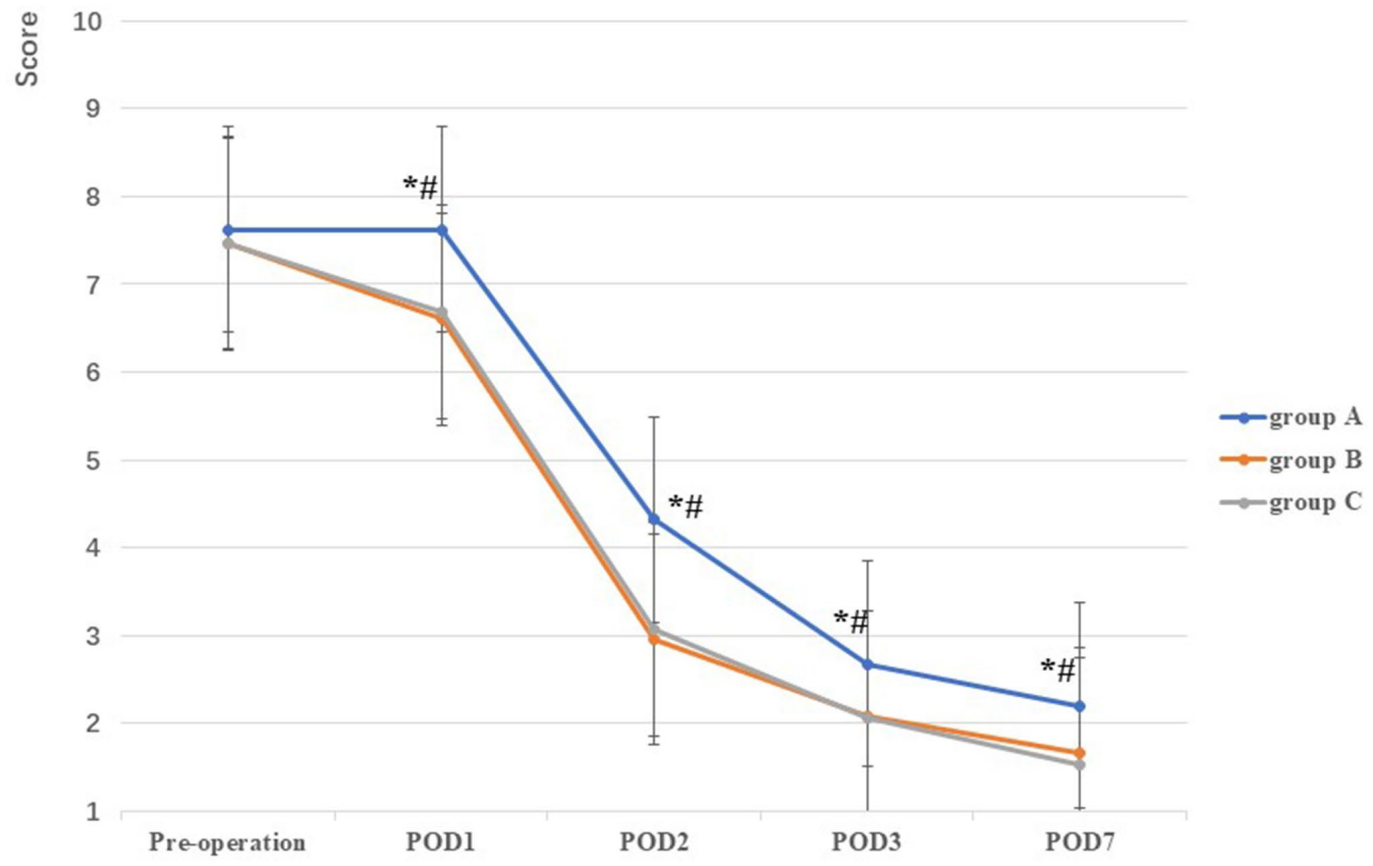
Comparison of VAS among the three groups at different stages. *: A vs B, *p*<0.05; #: A vs C, *p*<0.05, Δ: B vs C, *p*<0.05.

There was no significant difference in VAS among the three groups prior to TKA (*F* = 1.00, *p* = 0.37). Compared with group A on POD1, POD2, POD3, and POD7 (7.76 ± 0.59, 4.32 ± 0.78, 2.68 ± 0.59, and 2.20 ± 0.57, respectively), VAS scores in groups B (6.60 ± 0.67, 2.96 ± 0.67, 2.08 ± 0.57, and 1.66 ± 0.63, respectively) and C (6.68 ± 0.79, 3.08 ± 0.63, 2.06 ± 0.79, and 1.54 ± 0.65, respectively) were lower, and the difference was statistically significant (all *p* < 0.001). However, no statistically significant difference was observed between groups B and C on POD1–POD3 and POD7 (all *p* > 0.05) (Table 2; Figure 4).

### 3.4 Joint swelling

Circumference variations on POD3 and POD7 were significantly lower for groups B (4.21 ± 0.48 and 3.72 ± 0.33, respectively) and C (4.23 ± 0.32 and 3.70 ± 0.31, respectively) than for group A (4.50 ± 0.49 and 3.98 ± 0.32, respectively) (all *p* < 0.001). No statistically significant difference was observed between groups B and C (*p* > 0.05) on POD3 and POD7 (Table 2).

### 3.5 PLOS

While PLOS was significantly shorter for groups B (10.12 ± 0.56) and C (10.12 ± 0.59) compared with group A (10.76 ± 0.89) (*p* < 0.001), no statistically significant difference was observed between groups B and C (*p* = 0.47).

### 3.6 Vit D levels

**Figure 5:**
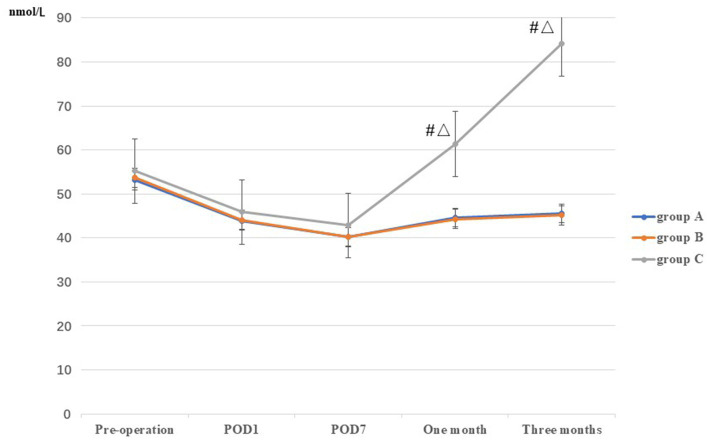
Comparison of Vit D among the three groups at different stages. *: A vs B, *p*<0.05; #: A vs C, *p*<0.05, Δ: B vs C, *p*<0.05.

No significant difference in Vit D levels were observed among the three groups prior to TKA, or on POD1 and POD7 (*p* > 0.05). At 1 and 3 months postoperatively, the levels of Vit D in group C (61.34 ± 6.86 and 84.20 ± 5.44, respectively) were higher than those in groups A (44.56 ± 7.53 and 45.52 ± 6.69, respectively) and B (44.30 ± 7.20 and 45.14 ± 6.93, respectively), and the difference was statistically significant (*p* < 0.05). However, no statistically significant difference was observed between groups A and B (*p* > 0.05) (Table 2; Figure 5).

### 3.7 Degree of satisfaction

As shown in Table 3, four patient satisfaction grades were reported: excellent, good, minimal, and poor, with grades of excellent and good considered to indicate patient satisfaction with treatment. The degrees of satisfaction in groups A, B, and C were 70, 80, and 78%, respectively. No significant differences were observed among the three groups (Table 3).

**Table 3:** Satisfaction degree among the three groups.

	A	B	C	D
Group A	28	7	7	8
Group B	32	8	5	5
Group C	32	7	6	5
χ^2^/*P*	1.77/0.94			
χ^2^/*P*1	1.36/0.72			
χ^2^/*P*2	1.04/0.79			
χ^2^/*P*3	0.16/0.98			

P, A vs B vs C; P1, A vs B; P2, A vs C; P3, B vs C. A, wonderful; B, good; C, not bad; D, terrible.

### 3.8 Postoperative complications

In our study, the incidence of deep vein thrombosis in groups A, B, and C were 3/50 (6.0%), 2/50 (4.0%), and 2/50 (4.0%), respectively, and the difference was not statistically significant. No patients had any complications in relation to frostbite, surgical site infection, or Vit D intoxication (Table 4).

**Table 4:** Postoperative complications among the three groups.

	Group A	Group B	Group C	*p*
DVT	3/50	2/50	2/50	0.86
Skin frostbite	0	0	0	–
SSI	0	0	0	–
Vit D intoxication	0	0	0	–

DVT, deep vein thrombosis; SSI, surgical site infection; Vit, vitamin.

## 4 Discussion

This study showed the AKSS on POD7 and at 1 month; the VAS on POD1, POD2, POD3, and POD7; thigh swelling on POD3 and POD7; knee joint ROM on POD3, POD7, and at 1 month; and POLS in group B were significantly higher than those in group A (*p* < 0.05). We concluded that cyclic cryotherapy could reduce postoperative pain and improve function post-TKA, and promote early rehabilitation. AKSS and ROM values at 1 and 3 months postoperatively in group C were significantly higher to those in group B (*p* < 0.05). We concluded that cyclic cryotherapy combined with Vit D could facilitate early rehabilitation post-TKA at 1 and 3 months. However, patient satisfaction scores did not improve significantly.

TKA is an effective surgical intervention for end-stage KOA, and shows long-lasting clinical and structural improvement for the management of severe OA, providing better overall improvements in function, mobility, pain, and health-related quality of life (18). However, patients in the immediate postoperative period frequently experience acute pain, severe oedema, and a reduced ROM, and attention should be paid to these issues. While patient satisfaction scores have improved (19), our findings indicate that further efforts are needed to reduce postoperative pain, improve knee function, and promote early rehabilitation post-TKA (20).

Cryotherapy was commonly first used for soft tissue injuries in sports medicine, and it has subsequently been widely used to facilitate good detumescence and for its analgesic effect (21). TKA requires extensive dissection of soft tissue and synovectomy, which inevitably result in extensive soft tissue injury; therefore, investigating the role of cryotherapy post-TKA is likely to be of value. We used a novel cryotherapy technique to apply intermittent cold to the knee. Some studies have shown that cryotherapy can appreciably reduce intraarticular temperature, especially in the knee, and reduce blood flow through vasoconstriction, the local inflammatory reaction, postoperative blood loss, thigh swelling, pain transmission, and the length of hospital stay (7, 22). Compared with standard cryotherapy (ice/gel pack application), cyclic cryotherapy devices have been developed that are more efficient as they maintain a steady low temperature for an extended period of time (23, 24). Thus, cyclic cryotherapy could accelerate rehabilitation time post-TKA through reducing inflammation, pain, and swelling.

We used the cyclic cryotherapy technique, which involved intermittent pressure cold compression on patients within 1 week postoperatively. Our findings indicated that the AKSS, pain VAS, degree of thigh swelling, ROM, and PLOS in group B were significantly better than those in group A in the early postoperative period, and the difference was statistically significant (*p* < 0.05). Moreover, cyclic cryotherapy did not increase the incidence of deep vein thrombosis post-TKA during the perioperative period. This suggests that the cyclic cryotherapy technique is helpful in controlling early postoperative pain and swelling, improving joint function, and in significantly promoting accelerated recovery post-TKA. In terms of its mechanism of action, cyclic cryotherapy reduces the temperature of surrounding tissues and the cell metabolic rate, thus reducing tissue hypoxia. At the same time, this local cooling also contracts capillaries, as well as reducing exudation, leukocyte adhesion, and the production and release of inflammatory factors; thus, relieving pain (21). In addition, cyclic cryotherapy has been found to increase intraarticular pressure, contract capillaries, reduce the flow of fluid in the tissue and reduce exudation to reduce edema (21, 25).

Vit D is a fat-soluble steroid hormone precursor, which functions to improve osteoblastic activity, increase bone mineral density, and may transform articular cartilage (11, 12). Currently, Vit D-related studies have focused mostly on KOA, with limited research on improving functional rehabilitation post-TKA, and with no uniform conclusions reported. Traven et al. (26) retrospectively analyzed 126 patients who had undergone hip and knee revision surgery. They reported no association between a low level of Vit D and the risk of 30-day readmission; however, an association between low levels of Vit D and an increased risk of complications and periprosthetic joint infection at 90 days was observed, as well as a lower joint function score (*p* < 0.05). Allain et al. (27) reported similar findings. Hwang et al. (15) reported that Vit D deficiency was very common in older adult women in their study. They found that preoperative Vit D deficiency had no significant effect on knee joint function in the short-term postoperatively (*p* > 0.05) based on a follow-up of 1,013 patients with TKA for >12 months, which was not consistent with our results. Some studies have reported that Vit D plays a role in improving muscle strength, while other studies have also shown that Vit D plays an important role in reducing chronic inflammation and relieving chronic pain (28–30). However, no studies have investigated the application of Vit D combined with cryotherapy in the TKA perioperative period. In group C, we administered cyclic cryotherapy in combination with Vit D. We intermittently applied a cold compress to the patients’ knees within 1 week postoperatively, and 200,000 units of Vit D were administered on POD1, followed by 800 units of Vit D as a daily supplement for 3 months. The results showed that the AKSS and the ROM in group C were significantly improved compared with those in group B at 1 and 3 months postoperatively, and the difference was statistically significant (*p* < 0.05). Our findings suggest that cyclic therapy combined with Vit D can help to improve joint function at 1 and 3 months and accelerate recovery post-TKA, and our findings are consistent with those in previous studies (31, 32). Notably, the levels of Vit D in groups A and B decreased briefly postoperatively and then slowly recovered, but still failed to recover to preoperative levels at 3 months postoperatively. This may be related to the loss of bone mass and activity resulting from surgery, and a decrease of Vit D supplementation and absorption owing to poor diet in the short-term postoperatively. In addition, no significant difference in the AKSS was observed on POD7, in the VAS on POD1– POD3 and POD7, in thigh swelling on POD3 and POD7, in ROM on POD3 and POD7, and in PLOS between groups B and C, which also indicates that the absorption and utilization of Vit D is a slow process. Moreover, no significant improvement in pain was noted in the acute phase, which was consistent with the level of Vit D. Based on these findings, we consider that cyclic cryotherapy combined with Vit D is beneficial in promoting continuity of rehabilitation after TKA at 1 and 3 months. Shin et al. (14) conducted a prospective study on 92 patients and followed up for 3 months, and also found that patients with Vit D deficiency had poor joint function recovery post-TKA, and the difference was statistically significant (*p* < 0.05). However, some studies have reported that the level of Vit D had no significant improvement effect on functional rehabilitation following joint replacement (15, 28, 33). Therefore, the effects of Vit D levels on postoperative rehabilitation in relation to TKA require further investigation.

No statistically significant difference in patient satisfaction was observed among the three groups; however, low satisfaction rates post-TKA have also been reported elsewhere (34, 35). One explanation for the patient satisfaction results is that we evaluated patient satisfaction prior to discharge. Another possible reason may be related to the ongoing effects of cyclic cryotherapy and Vit D. Further efforts are needed to enhance patient satisfaction rates post-TKA at our institution.

This study had some limitations. Only assessing patients up to 3 months of follow-up might have restricted our capacity to assess long-term safety profiles and the efficacy of cyclic cryotherapy and Vit D supplementation. Given this was a small-sample single-centre study, the reliability of our findings should be validated using larger samples in multicentre studies. Moreover, a single-blind randomized controlled trial may cause subjective bias in the results. We could not confirm whether cyclic cryotherapy was superior to standard cryotherapy. We evaluate satisfaction of the patients only at discharge and ignored that at 3 months, which may bring some bias in patient satisfaction. The effects of cryotherapy combined with Vit D on inflammation were not investigated; however, we intend to investigate this in future research.

## 5 Conclusion

Cyclic cryotherapy post-TKA showed short-term advantages in terms of the AKSS, VAS, thigh swelling, ROM, PLOS, and accelerated rehabilitation, but it did not improve patient satisfaction scores. Cyclic cryotherapy combined with Vit D also improved the AKSS and ROM at 1 and 3 months postoperatively.

## Data availability statement

The raw data supporting the conclusions of this article will be made available by the authors, without undue reservation.

## Ethics statement

The studies involving humans were approved by the People's Hospital of Guangxi Zhuang Autonomous Region. The studies were conducted in accordance with the local legislation and institutional requirements. The participants provided their written informed consent to participate in this study.

## Author contributions

FL: Writing – original draft, Conceptualization, Data curation, Investigation, Software. YM: Conceptualization, Data curation, Investigation, Writing – original draft, Methodology. XH: Project administration, Resources, Writing – review & editing. KS: Formal analysis, Project administration, Resources, Conceptualization, Writing – original draft. BL: Resources, Methodology, Supervision, Writing – original draft. DY: Funding acquisition, Writing – original draft, Writing – review & editing.
